# Risk Factors for Persistent Infection of Non-Typhoidal Salmonella in Poultry Farms, North Central Nigeria

**DOI:** 10.3390/antibiotics11081121

**Published:** 2022-08-18

**Authors:** Abdullahi O. Sanni, Joshua Onyango, Abdulkadir Usman, Latifah O. Abdulkarim, Annelize Jonker, Folorunso O. Fasina

**Affiliations:** 1 Department of Veterinary Tropical Diseases, University of Pretoria, Onderstepoort, Pretoria 0110, South Africa; 2 Agro-Processing, Productivity Enhancement and Livelihood Improvement Support (APPEALS) Project, Lokoja 260101, Kogi State, Nigeria; 3 Harper and Keele Veterinary School, Harper Adams University, Newport TF10 8NB, UK; 4 Department of Animal Production, Federal University of Technology, Minna 920101, Niger, Nigeria; 5 ECTAD Food and Agriculture Organization of the United Nations, Nairobi 00100, Kenya

**Keywords:** non-typhoidal Salmonella, poultry, risk factor, Nigeria, fowl typhoid, pullorum disease

## Abstract

Salmonellosis is a bacterial zoonosis causing an array of health conditions. Non-typhoidal salmonellosis (NTS) has a discrete adaptation to certain animals; in poultry, pullorum and fowl typhoid are its primary disease manifestations. The diseases are prevalent in Nigerian poultry and have been well-studied in Nigeria, but less so in North Central Nigeria (NCN). Using field sampling, laboratory methods and a semi-structured questionnaire for 1000 poultry farms in NCN, we explored the incidence and risk factors for the persistence of NTS infection in poultry. Approximately 41.6% of the farms had experienced NTS over the last 18 months. Farm experience of NTS moderately predicted awareness of salmonellosis. Increasing stock in smallholder farms, self-mixing of concentrate on the farm, usage of stream water, pen odour, non-adherence and partial adherence of farms to recommended poultry vaccination against pullorum and fowl typhoid and lack of and non-adherence to biosecurity were identified risk factors that increased the odds of NTS infection in poultry. Antibiotic use practice may have reduced the isolation rate of NTS, yet NTS continues to challenge poultry farms in Nigeria. Identified risk practices must be mitigated intentionally and biosecurity and hygiene must be improved to reduce the burden of NTS.

## 1. Introduction

Fowl typhoid and pullorum disease are bacterial infections (salmonellosis) found in farmed poultry caused by the *Salmonella enterica* subspecies *enterica* serovars *Gallinarum* biovars *Gallinarum* and *Salmonella enterica* subspecies *enterica* serovar *Gallinarum* biovar *Pullorum*, respectively, and they are widely distributed globally [1,2]. Recent evidence has also suggested a tendency towards increasing antimicrobial resistance in strains of these organisms obtained from poultry [3,4,5]. Although its eradication is possible, and this has been largely achieved in many commercial poultry in developed countries in Western Europe, the United States of America (USA), Canada, Australia and Japan, its eradication in developing countries, particularly in Africa, Asia and South America, remains debatable [6,7,8].

Salmonellosis is a bacterial zoonoses with considerable public health impacts, and it can be caused by typhoidal and non-typhoidal Salmonella organisms, including those mentioned above [8,9]. According to FoodNet surveillance data, Salmonella causes more disease burden in humans than any other foodborne pathogen, and globally, it causes up to 20 million human cases annually [8,9,10]. In the USA alone, salmonella-contaminated poultry is responsible for an estimated loss of USD 2.5 billion annually, or the loss of 15,000 QALYs in annual disease burden [9,10]. This considerable burden of disease is caused by food handling and preparation problems in food service and retail settings, some of which may have been associated with contaminations along the food chain [5,9,10].

Non-typhoidal Salmonella (NTS) refers to the infection produced by all serotypes of Salmonella except for the typhoidal and paratyphoidal groups. Although there have been at least 2463 serotypes of Salmonella found to date (over 2500 by other estimates) [11,12,13,14], the laborious traditional phenotypic serotyping method is still popular. It is challenging because it involves more than 150 specific antisera and expert interpreters to analyse the results [12]. In recent times, proposals for genome-based Salmonella serotyping and microarray methods have been made [12,15]. The symptoms of NTS in humans include diarrhoea, vomiting and abdominal cramps, which develop 12 to 72 h after infection. NTS has a discrete adaptation to certain animals, such as the adaptations of *Salmonella Choleraesuis* to pigs, *Salmonella Dublin* to cattle, *Salmonella Abortusovis* to sheep and *Salmonella Gallinarum* (*Salmonella enterica* subspecies *enterica* serovars *Gallinarum* biovars *Gallinarum*) and *Salmonella Pullorum* (*Salmonella enterica* subspecies *enterica* serovar *Gallinarum* biovar *Pullorum*) to poultry [2,11,16,17].

In Nigeria, the burden of zoonotic salmonellosis is unknown in humans or poultry; however, significant research has been produced on salmonellosis in poultry [3,18,19,20,21,22,23,24,25]. However, these studies have been concentrated in the extreme north and the southern belt of the country. North Central Nigeria (NCN), which connects the southern belt of the country, where most of the commercial poultry activities occur, with the north, where most of the indigenous poultry populations predominate, has been less investigated. It is estimated NCN had a significant poultry population in excess of 44,789,854 in 2020 [26], and it is the producer of the majority of meat and eggs supplied to the Federal Capital Territory and its neighbourhood. There is therefore a need to carry out a series of empirical studies, including one on the risk factor for continuing infections of poultry farms with Salmonella in North Central Nigeria, to bridge the existing knowledge gaps that exist in salmonella studies in Nigeria in order to inform policy aimed at reducing the burden of this bacteria zoonosis. The goals of this study were (i) to investigate the prevalence of non-typhoidal Salmonella in the poultry farms in North Central Nigeria, and (ii) to explore potential risk factors in commercial and backyard poultry farms in North Central Nigeria.

## 2. Results

**Figure 1:**
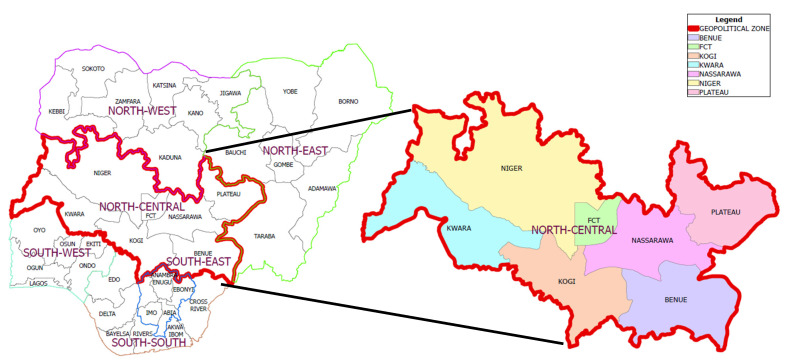
Map of Nigeria with a call-out map of the North Central zone.

This work covered the six states of the North Central zone of Nigeria (Kogi, Niger, Nasarawa, Kwara, Benue and Plateau) and the Federal Capital Territory (FCT) (Figure 1). One hundred and fifty (150) samples were collected from three local government areas (LGAs) (50 farms per LGA) in every state surveyed except in the state of Plateau, where 100 samples were collected from two LGAs (n = 1000). In the period under consideration (≤18 months, September 2020–March 2022), 416 farms (41.6%) experienced non-typhoidal Salmonella (NTS)—*S. enterica*, as confirmed by veterinary laboratory evaluations and reports, and based on clinico-pathological evaluations of the farms. Apart from *Salmonella enterica*, *Klebsiella pneumoniae* was detected in 92.9% of the samples, *Lactobacillus bulgaris* was found in 0.9% of the samples, *Salmonella arizonae* was detected in 0.2%, *S. paratyphi* in 1.9% and *S. typhi* in 2.3% of all samples (Table 1). A total of 392 of the 416 *S. Enterica*-positive samples (94.5%) exhibited mixed infections with *Klebsiella pneumoniae*, *Lactobacillus bulgarius*, *S. arizonae* and/or *S. paratyphi*.

The percentages of farmers with ≤2 years, >2–≤4 years, >4–≤6 years and >6 years of experience were 22.4%, 31.9%, 23.9% and 21.8%, respectively. The majority of the interviewed farmers had a tertiary education (50.8%), and only 49.2% had other forms of education, up to the secondary level. Among the farms surveyed, 44.4% practiced broiler operations, 22.5% carried out layer operations, and 29.4% carried out mixed operations (layers and broilers on the farm) (Table 2). Details of other descriptive statistics on all farm- and field-level data are described in Table 2.

Using pairwise correlations, most of the risk- and management-related variables evaluated against the experience of Salmonella in farms were weakly or negatively correlated, except for the awareness of Salmonellosis (NTS) as a potential zoonosis, which was moderately correlated with the experience of Salmonella in poultry farms (Table 3). The higher the number of poultry chickens on the farm, the higher the odds of NTS on the farms. In particular, having between 500 and 1000 chickens on the farm increased the risk of infection three-fold (*p* < 0.001), and having >1000 chickens increased the risk of persistent infection by ≈4-fold (*p* < 0.001) (Table 4). Farmers who self-mixed concentrate on the farm had a 2-fold-increased risk of persistent NTS infection (*p* < 0.001), and the use of stream water produced the same odds (*p* < 0.01). Chickens in poultry cages had 2-fold-increased odds of persistent NTS infection (*p* < 0.001), and non-adherence of farms to recommended poultry vaccination against pullorum and fowl typhoid increased the odds of NTS infection by >7-fold (*p* < 0.001), and even partial adherence increased the risk over four-fold (*p* < 0.001) (Table 4). Farmers who were not implementing and applying the principles of biosecurity strictly had 2-fold-increased odds of NTS infection on their farms (Table 4). The laying stock was approximately two-fold as likely to be infected with persistent NTS compared with short-cycled broilers (*p* = 0.002). Finally, farms with no pen odour were 8-fold less likely to experience NTS infection compared with pens with a persistent odour (*p* < 0.001) (Table 4). 

**Figure 2:**
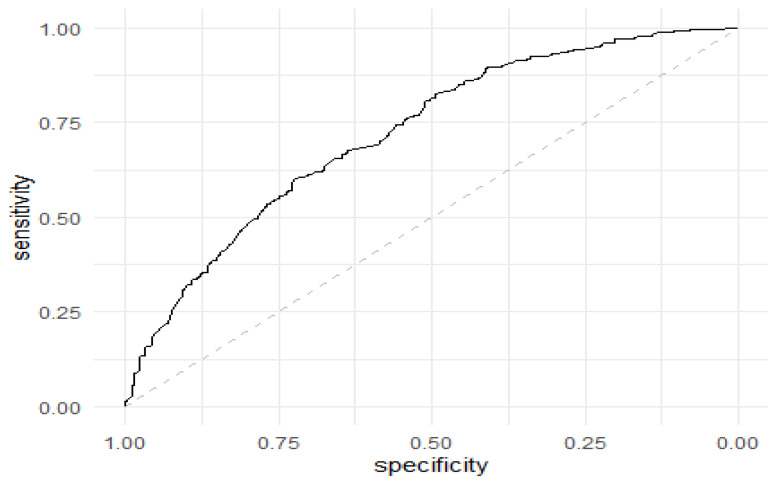
Receiver operating characteristics of risk factor model for persistent infection of non-typhoidal Salmonella on poultry farms, North Central Nigeria. The ROC curve (solid curve) performed better than the diagonal line (dotted line) at 0.72, a reflection that the performance of the diagnostic test that is better than chance level.

According to the multivariable logistic regression model, the higher the number of poultry chickens on the farm, the higher the odds of NTS on the farm (500–1000 chickens, OR = 2.20, *p* < 0.001; >1000 chickens, OR = 2.17, *p* = 0.004), whereas dug-up wells reduced the odds of infection by half (OR = 0.57, *p* = 0.01), and use of stream water as a source of drinking water for poultry birds increased the odds of NTS infection by >3-fold (*p* = 0.005) (Table 5). Of note, both the partial and non-adherence of farms to the recommended poultry vaccination against pullorum and fowl typhoid increased the odds of NTS infection in the poultry farms five-fold for each (Table 5). The Hosmer–Lemeshow goodness of fit = χ2 = 2.58; *p* = 0.96; Akaike information criterion (AIC) = 945.52; Area under curve (receiver operating characteristics (ROC)) = 0.72 (Figure 2).

## 3. Discussion

The total burden of zoonotic salmonellosis in humans or poultry in Nigeria is unknown [3,18,19,20,21,22,23,24,25]. NCN serves the Federal Capital Territory and burgeoning neighbourhoods with food, including animal-sourced food. In this regard, this work is timely and meets the need to prevent food-borne zoonoses and related infections in the North Central belt of Nigeria (Figure 1; [28]). In this study, bacteria culture and phenotypic and biochemical characterization were used as the basis for identification and confirmation of non-typhoidal Salmonella. Culture and phenotypic and biochemical characterization have been confirmed as very sensitive and specific for the identification of NTS, and they compare favourably with PCR and ELISA [2,29,30].

Although Klebsiella pneumoniae and other isolated organisms were incidental findings in this study, a recent report has documented the prevalence of Klebsiella pneumoniae in 41.7% of healthy poultry [31]. Klebsiella pneumoniae is an opportunistic pathogen, and a commonly isolated cause of nosocomial infections in humans, together with five other bacteria, referred to as the ESKAPE pathogens (Enterococcus faecium, Staphylococcus aureus, Klebsiella pneumoniae, Acinetobacter baumannii, Pseudomonas aeruginosa and Enterobacter spp) [32]. It is unsurprising that it was the most isolated pathogen in this study because other studies have confirmed that K. pneumoniae may cause disease in poultry, and may co-habit with Salmonella spp. and be resistant to extended-spectrum beta-lactamase (ESBL) and carbapenemase antimicrobials, some of which may be passed onto the human food chain, causing resistant pathogens in humans [33,34,35]. In Trinidad and Tobago, 23 different Salmonellae have been found in broiler production with a prevalence of between 8.9 and 20.5% [5]. Similarly, in a recent survey in Great Britain involving 23 commercial broiler hatcheries, a prevalence of between 0 and 35% was obtained for the chick-handling areas, hatcher areas, macerator areas, tray wash/storage areas, external areas and other waste-handling areas, which are more contaminated in hatchery operations [36]. 

The prevalence of NTS in the surveyed smallholder poultry farms was 41.6% based on laboratory findings, and following clinico-pathological evaluations over a period of 18 months. This prevalence was similar to previous findings from Nigeria by Jibril et al. [37] and Fagbamila et al. [21,38], who previously reported a farm-level prevalence of 47.9% and 43.6% in Nigeria. We obtained samples from broiler and layer farms but did not consider the hatcheries and parent/grandparent farms. These latter farms need special permission to access and may have to be considered separately in a specialized study. Such a study may ascertain whether there are linkages between hatcheries and parent/grandparent farms on one hand and commercial farms on the other hand, particularly in the transmission and dispersal of NTS in the poultry food chain [39,40,41]. The weak correlations among the risk factors observed in the study meant that most of the factors considered cannot predict other factors and anthropogenic influence may affect how each factor plays a role. However, the awareness of Salmonella was moderately correlated with having experienced Salmonella on the farm (Table 3), an indication that previous or current experience of NTS on the farm is a positive predictor for awareness of Salmonella infection.

In our observation, the source of water and litter materials varied from farm to farm, and there was wide disparity in adherence to sanitary practices (Table 2). These sources, especially when they come from untreated sources, predispose farms to infection. Extension agents were confirmed as significant sources of knowledge for the farmers in this study (86%), and access to veterinary professionals and paraprofessionals was not always guaranteed (33.9%); thus, extension agents could be used as agents of change in risk communication and community engagement with regard to awareness and targeted messaging to farmers about the risk of poultry salmonellosis. For effectiveness and efficiency, the extension agents will need to be trained appropriately in relevant animal health matters, as anecdotal evidence revealed that most of the extension agents were skewed towards plant production and health. 

It should be noted that the pathogen population increases with farm intensification and crowding of poultry per unit space [42]; thus, it is not surprising that the more chickens there were on the poultry farms, the higher the odds of infection with NTS were (Table 4). Similarly, the use of stream water as a source of drinking water for chickens increased the risk of infection with NTS by 3-fold. It is highly likely that stream water is perpetually contaminated and its use without treatment will predispose poultry farms to infection. Farms are encouraged to pretreat stream water for use on their farms. While it is expected that ground water would increase the risk [43], the well water decreased the risk by half (Table 4). We are aware that most dug-up well are regularly treated with chlorine, and this may have positively influenced the reduction in the burden of risk observed in this case. We confirmed that the odds of NTS infection through feed was slightly high. Other workers [44] have recently confirmed that the incidence of NTS (S. enterica) in poultry feed and feed ingredients may range from 0 to 78%, and these may serve as a source of infection on poultry farms. Pen odour increased the risk by almost two-fold, which is more an indication of the poor hygiene practices and poor litter management on the farm rather than a risk factor itself. It is therefore important to advocate for better litter management and good farm hygiene practices to mitigate against infection with NTS. 

Most importantly, the non-adherence to pullorum and fowl typhoid vaccinations (AOR = 5.2) and partial adherence to vaccinations (AOR = 5.1) both significantly increased the risk of infection with NTS infection in poultry. It is confirmed that vaccination against Salmonella infection in poultry is not capable of eradicating infection from flocks but only offer an extra layer of protection, increase the threshold for infection, reduce the level of shedding of the organism and reduce vertical transmission in poultry, thus preventing contamination of hatching or table eggs [2]. The advantage of such vaccinations in reducing the risk of NTS in smallholder poultry farms is obvious. However, we advocated for support with other practices as emphasized in the standard protocol for control and eradication of NTS in poultry [2]. In this work, only 64.4% of farms adhered to vaccination protocol, and only 55.5% of the farmers implemented and adhered to biosecurity practices, and only 27% of the farmers adhered to the protocol of culling of infected flocks. However, a number of surveyed farmers continued to practice non-recommended practices against NTS eradication, including the administration of antibiotics (0.7%), vaccinations (36.9%), a combination of antibiotics and vaccination (11.5%) and the sale of infected poultry to consumers (13.2%). These practices are likely to further horizontal transmission of NTS to other farms and increase the risk for zoonosis. (Table 2 and Table 4). 

We are aware that this work is subject to some limitations. Firstly, complete serotyping of all classified positive cases was not performed, as this may have revealed all the serotypes of Salmonellae harvested over the 18 -month period. While full serotyping may be beneficial research-wise, and to inform policy, it should be noted that serotyping for Salmonella is a relatively expensive procedure, and smallholder poultry farms may consider this too burdensome to bear financially. Perhaps the authorities may consider covering the full cost of diagnosis for smallholder farms with cases of NTS. Secondly, several laboratories were utilized to determine the positivity for NTS, and not all farm cases were submitted for laboratory evaluation, some of which may have been salmonellosis. This potentially exposed the study to misclassification, a situation that may have increased/decreased the total prevalence determined in the study. 

## 4. Materials and Methods

### 4.1. Selection of States and Sampling Sites

The states in this geopolitical zone include: Kogi, Niger, Nasarawa, Kwara, Benue, Plateau and the FCT (Figure 1). The selection of this study site was informed by the lack of empirical data sources on non-typhoidal Salmonella (NTS) from North Central Nigeria (NCN), and the need to aggregate the risk factors for persistence of non-typhoidal Salmonella in poultry farms in NCN. 

### 4.2. Development of Questionnaire and Training of Data Collectors

Through a literature review and probing questions to veterinarians and animal health assistants, a list of previously identified risk factors for Salmonella in poultry in Nigeria was developed ([37,45]; Supplementary Material File S1). A semi-structured questionnaire was prepared based on this list of identified risk factors and drivers of NTS infection on farms. Although the questionnaire was prepared in English, and approximately 90% of all respondents had at least a secondary level of education, respondents were allowed to choose a convenient language for communication during the interview. All communication was in the English language or local dialects, as selected by the respondent, to enable the respondents to communicate effectively or provide detailed inputs. The questionnaire targeted data on location, demographics, years of experience, type of management and chickens kept, housing and farm environment details, awareness of Salmonella, case and mortality patterns and some economic variables, as well as access to professional support.

Hired research assistants (HRAs/data collectors) (n = 21) were recruited from the localities of the sampling sites in each of the states. The lead researcher (AOS) organized a training session for the HRAs on the objectives of the study, how to avoid bias during the field data collection and how to include internal quality control to enhance data validity. Five of the trained HRAs/data collectors conducted the role play exercise and served as respondents. Feedback from the role play exercise was used to improve the questionnaire. All questions were checked for consistencies, avoidance of ambiguity and misinterpretation. The pretested questions were printed in hard copies for the use of data collectors in the field.

### 4.3. Field Sampling and Laboratory Analysis

The maximum number of poultry farms was targeted for sampling per each state (n = 150 × six states = 900 samples, except for the state of Plateau, where 100 farms were visited; total = 1000). On each farm, up to five freshly voided faecal samples were pooled and collected in a sterile sample container. Pooling of each sample per farm was considered because a farm is considered as an epidemiological unit and a single case of salmonellosis on a farm makes the farm positive in this study. While samples were collected in sterile sample containers, a lead person (typically, the farm manager, farm owner or his/her designated assistant) was interviewed using the pretested questionnaire. The farms were randomly selected and recruited once they determined to qualify for the definition of a poultry farm, without bias regarding the bird types available on the farm or the farm size. All samples were transported on ice to the laboratory, and a total of 1000 samples and 1000 questionnaires were collected. The preferred sample was the freshly voided faeces or faeces collected directly using cloacal swab/massage. In a few cases, other samples (swabs of organs and tissues) were picked from dead carcasses (n = 12) [2], and were identified using the bacterial culture methods described below at the STEP-B laboratory of the Federal University of Technology Minna, Niger, and Central Research and Diagnostic Laboratory, Ilorin. All sample collections in live chickens were preceded by the presentation of ethical approval document approved by the Research Ethics Committee (REC) of the Federal University of Technology, Minna, Nigeria (Approval number: 000030).

### 4.4. Bacteriological Culture and Phenotypic and Biochemical Characterization

Collected and transported faecal swabs and organ samples were macerated in peptone water, and cultured for identification as previously described [2,46]. Briefly, approximately 25 g of each sample was weighed and added to 225 mL of 0.1% peptone water, and incubated overnight at 37 °C. The overnight-incubated suspension was transferred (0.1 mL of each to 10 mL of Rappaport-Vassiliadis Soy Peptone (RVS) Broth) (Merck, Darmstadt, Germany) and re-incubated overnight at 41.5 °C. Following the incubation, samples were cultured on Xylose Lysine Desoxycholate (XLD) agar (Merck, Germany) and incubated again overnight at 37 °C. Red colonies with a black centre were subcultured in nutrient agar (NA) (Merck, Germany) to perform Gram staining and biochemical tests [46]. Colonies were Gram-stained for identification, and biochemical characterization was performed for confirmation [2,46,47].

### 4.5. DNA Extraction and Polymerase Chain Reaction

Following bacteriological culture, selected bacterial-culture-positive isolates were subjected to further molecular characterization, as described here. DNA was extracted using the protocol stated by Zhang et al. [47]. The extracted DNA was processed for PCR using the 16S rRNA gene PCR forward and reverse primers: (27F, 5′-AGAGTTTGATCMTGGCTCAG-3′ and 1525R, 5′-AAGGAGGTGATCCAGCC-3′) and 0.3 units of Taq DNA polymerase (Promega, Madison, WI, USA). PCR was carried out in a GeneAmp 9700 PCR System Thermal cycler (Applied Biosystem Inc., Foster City, CA, USA) using the predefined PCR profiles (initial denaturation at 94 °C for 5 min; followed by 30 cycles at 94 °C for 30 s, 50 °C for 60 s and 72 °C for 1 min 30 s; a final termination at 72 °C for 10 min; and chilled at 4 °C) [22,46]. The final PCR product was electrophoresed on the 1.5% agarose gel using a 100 bp molecular weight ladder as a marker.

### 4.6. Definition of Case and Control Farms

For the purpose of risk factor evaluation, a case farm was defined as a poultry farm from which a biological sample collected from a suspected/unsuspected clinical case, tested in the laboratory according to the protocol mentioned above, and was consistently positive according to the test methods (culture and biochemical confirmation) in accordance with the international regulations for confirmed positive cases of poultry salmonellosis (fowl typhoid and pullorum diseases) [2]. Alternatively, poultry farms that had also experienced salmonellosis non-typhoidal Salmonella (NTS) within the period under consideration (≤18 months, equivalent to the maximum period for the current cycle of stocking of poultry chickens), and had been confirmed both clinico-pathologically and through laboratory confirmation, were included as case farms. For this work, a total of 416 case farms were found to have experienced NTS and tested positive for poultry salmonellosis in the last ≤18 months. A control farm was described as a farm where a sample was collected and tested as described for the case farm above but was negative according to all test protocols. Such farms must have been negative according to clinico-pathological as well as laboratory diagnostic tests. A total of 584 farms had not experienced poultry salmonellosis in the last batch of chickens present on their farms (≤15 months).

### 4.7. Statistical Analysis

Data were cleaned in Microsoft Excel 2018 and imported to Stata v 15 (Stata Corporation, College Station, 4905 Lakeway Dr., TX, USA) for analysis. Initially, we conducted descriptive statistics for all farm and collected field-level data to determine their proportions, standard errors (SEs) and 95% confidence intervals (CIs95%) for each variable, using the method of Agresti and Coull [27]. Categorical variables were also summarized as proportions. The disease prevalence was computed as the number of farms reporting to have had NTS at the time of the study or in the past, divided by the total number of study farms as a percentage. We aggregated selected risk-related variables and ran comparisons using pairwise correlation to determine whether there were significant correlations among the variables. Since the observations were not independent, a logistic regression model was used to investigate the association between the various potential risk factors and the outcome variable (defined as a farm having experienced NTS or not, and confirmed through clinical and laboratory diagnosis). The predictor variables used in the analysis are listed in Table 2, Table 3 and Table 4. The effect of each independent variable was first run in the univariable logistic regression model. Variables associated with the outcome (non-typhoidal salmonella (NTS) infection) at *p* ≤ 0.2 were considered for inclusion in the multivariable logistic regression model. Independent variables were tested for pairwise associations, using a two-tailed chi-square test. The model was progressively simplified using the backward stepwise elimination method. Backward stepwise regression is a stepwise regression approach that begins with a full (saturated) model and at each step gradually eliminates variables from the regression model to find a reduced model that best explains the data. The stepwise approach is useful because it reduces the number of predictors, reducing the multicollinearity problem, and it is one of the ways to resolve overfitting. Variables that were found not to have strong evidence of an association, or a Wald test with a *p*-value (>0.05), were excluded one at a time with the least statistically significant excluded at each step. To check that the variables removed did not have a huge effect on the model, the log likelihood ratio test was calculated each time. 

The Hosmer and Lemeshow test goodness of fit test was used to show how well the data fit the model. Model discrimination was assessed by using the area under the receiver operating characteristic curve (AUROC). The AUROC was used to compare the goodness of fit of logistic regression models, where values for the measurement ranged from 0.5 to 1.0. A value of 0.5 indicated that the model was no better than chance at making a prediction about membership in a group, and a value of 1.0 indicated that the model perfectly identified those within a group and those not. At each stage of backward stepwise elimination, the models’ discrimination and overall fit was assessed. All analyses were carried out in Stata v 15 (Stata Corporation, College Station, TX, USA). A statistical significance level was set at *p* < 0.05.

## 5. Conclusions

NTS continues to challenge poultry farms in North Central Nigeria, and some risk factors contributing to farm infection have been identified. Farm practices must be mitigated intentionally, and biosecurity and hygiene must be improved in order to reduce the burden of NTS. Finally, full compliance with vaccination protocols against pullorum and fowl typhoid in poultry combined with other control measures will assist in eradicating infection with NTS from poultry flocks in Nigeria.

## Figures and Tables

**Table 1:** Descriptive statistics of cultured bacteria found in faecal samples collected from smallholder poultry farms, September 2020–March 2022, North Central Nigeria.

Isolates	Number	Percentage
*Klebsiella pneumoniae*	929	92.9
*Lactobacillus bulgarius*	9	0.9
*Salmonella enterica*	416 *	41.6
*S. arizonae*	2	0.2
*S. paratyphi*	19	1.9
*S. typhi*	23	2.3

* A total of 392/416 (94.5%) of the samples with *S. enterica* infection had mixed infections with *Klebsiella pneumoniae*, *Lactobacillus bulgarius*, *S. arizonae* and/or *S. paratyphi*.

**Table 2:** Descriptive analysis of the respondents’ variables for the incidence of non-typhoidal Salmonella in poultry farms, North Central Nigeria.

Variable * (n)	Categories	Proportion (%)	95% Confidence Interval
States (1000)	Kwara	15.00	12.78–17.22
	Nasarawa	15.00	12.78–17.22
	Kogi	15.00	12.78–17.22
	Niger	15.00	12.78–17.22
	Plateau	10.00	8.14–11.86
	Benue	15.00	12.78–17.22
	FCT	15.00	12.78–17.22
Experienced confirmed cases of salmonellosis in the last 18 months (1000)	No	58.40	55.27–61.48
	Yes	41.60	38.54–44.66
Gender (1000)	Male	56.90	53.83–59.97
	Female	43.10	40.02–46.17
Experience in years on poultry farms (1000)	≤2 years	22.40	19.81–24.99
	>2–≤4 years	31.90	29.01–34.79
	>4–≤6 years	23.90	21.25–26.55
	˃6 years	21.80	19.23–24.36
Educational level of the poultry farmer (1000)	Primary	8.80	7.04–10.56
	Secondary	38.10	35.08–41.12
	Tertiary	50.80	47.70–53.90
	Others	2.30	1.37–3.23
Type of poultry (1000)	Broilers	44.40	41.31–47.48
	Layers	22.50	19.91–25.09
	Others	3.70	25.28–4.87
	Mixed	29.40	26.57–32.23
Number of chickens (1000)	≤200	34.90	31.94–37.86
	201–500	27.50	24.73–30.27
	501–1000	25.90	23.18–28.62
	≥1000	11.70	9.70–13.70
Source/type of feed (999)	Concentrate	59.46	56.41–62.51
	Mix	23.72	21.08–26.37
	Self-compounded	16.82	14.49–19.14
Source of water for chickens (999)	Borehole	46.05	42.95–49.14
	Tap borne (municipal)	20.22	17.73–22.72
	Well	29.53	26.70–32.36
	Stream	4.00	2.79–5.22
	Other	0.20	0.07–0.48
Pen type (998)	Standard block	30.06	27.21–32.91
	Dwarf block	41.98	38.92–45.05
	Zinc type	24.64	21.97–27.33
	Others	3.31	2.20–4.42
System of management (1000)	Deep litter	64.20	61.22–67.18
	Battery cage	31.80	28.91–34.69
	Others	4.00	2.78–5.22
Type of litter material used (1000)	Sawdust	42.90	38.83–45.97
	Wood shavings	30.20	27.35–35.05
	Sand	11.70	9.70–13.70
	Cement floor	14.00	11.85–16.15
	Others	1.20	0.52–1.88
Litter management (1000)	Poor	65.20	62.24–68.16
	Fair	9.50	7.68–11.32
	Good	25.30	22.60–28.00
Pen odour (1000)	No	41.60	38.54–44.66
	Yes	58.40	55.34–61.46
Stocking density (chickens per square meter of available floor space) (998)	12–14	17.43	15.08–19.79
	14–16	18.24	15.84–20.64
	16–18	22.04	19.47–24.62
	18–20	11.52	9.54–13.51
	20 and above	6.71	5.16–8.27
	Unknown	24.05	21.39–26.70
Adherence to vaccination (1000)	No	8.10	6.41–9.79
	Yes	64.40	61.43–67.37
	Partial	27.50	24.73–30.27
Practiced biosecurity (1000)	No	11.40	9.43–13.37
	Yes	55.50	52.41–58.59
	Partial	33.10	30.18–36.02
Had previously heard of salmonellosis (1000)	No	34.90	31.94–37.86
	Yes	64.90	61.94–67.86
	Do not know	0.20	0.08–0.48
Experienced confirmed cases of salmonellosis in the last 1–2 years (1000)	No	30.90	28.03–33.77
	Yes	41.60	38.54–44.66
	Do not know	27.50	24.73–30.27
When salmonellosis or mixed infection was experienced on the farm, how was it handled? Or what protocol was used? (1000)	Antibiotics	0.70	0.18–1.21
	Vaccination	36.90	33.90–39.90
	Antibiotics combined with vaccination	11.50	9.52–13.48
	Culling	27.00	24.24–29.76
	Sales	13.20	11.10–15.30
	Others	10.60	8.69–12.51
	No response	0.10	0.09–0.30
Had the knowledge (awareness) of salmonellosis as a zoonotic disease (1000)	No	38.00	34.99–41.01
	Yes	60.80	57.77–63.83
	No response	1.20	0.66–2.11
Source of knowledge (1000)	Electronic media	11.00	0.45–1.75
	Print media	35.40	32.43–38.37
	Extension agent	86.00	6.86–10.34
	Vet/AHO	9.40	7.59–11.21
	Other farmers	26.10	23.37–28.83
	Hospital	15.80	13.54–18.07
	Other sources	3.60	2.44–4.76
Had previously taken samples to veterinary service (1000)	No	36.00	33.02–38.98
	Yes	62.10	59.09–65.11
	No response	1.90	1.20–2.97
Access to professional support (1000)	No	26.70	23.95–29.44
	Yes	33.90	30.96–36.84
	Not always	37.40	34.40–40.40
	Others	2.00	1.13–2.87

All analysis was conducted using the method of Agresti and Coull [27] and reported using the binomial Wald method. * Categorization of variables based on selected industry standards and the peer-reviewed literature (Table S1).

**Table 3:** Pairwise correlation of selected variables for incidence of non-typhoidal Salmonella on poultry farms, North Central Nigeria.

	Experienced Salmonella	Gender	Farming Experience in Years	Education Level	Type of Farms	No. of Chickens	Feed Source	Water Source	Management System	Litter Management	Pen Odour	Stocking Density	Adherence to Vaccination	Practice Biosecurity	Had Heard of Salmonella	Knowledge of Salmonella
Experienced Salmonella	1.000															
Gender	−0.003	1.000														
Farming experience in years	0.041	0.083 *	1.000													
Education level	0.017	0.032	0.234 *	1.000												
Type of farm	0.097 *	0.084 *	0.189 *	0.120 *	1.000											
No. of chickens	0.233 *	0.084 *	0.145 *	0.080 *	0.149 *	1.000										
Feed source	−0.156 *	−0.004	0.099	0.004	0.095 *	−0.079 *	1.000									
Water source	−0.172 *	0.009	0.090 *	−0.068 *	0.025	−0.157 *	0.257 *	1.000								
Management system	−0.125 *	−0.022	−0.014	0.008	−0.096	−0.237	0.100	0.136 *	1.000							
Litter management	−0.071 *	−0.051	−0.116 *	−0.151 *	−0.049	−0.108 *	0.177 *	0.136 *	0.044	1.000						
Pen odour	0.029	−0.005	0.003	−0.021	−0.007	0.014	0.075 *	0.232 *	0.086 *	0.152 *	1.000					
Stocking density	−0.110 *	0.011	0.063 *	−0.022	−0.063 *	−0.009	0.053	0.021	0.056	0.093 *	−0.006	1.000				
Adherence to vaccination	0.178 *	0.116 *	0.074 *	0.109 *	0.071 *	0.219 *	−0.237	−0.165 *	−0.059 *	−0.224 *	−0.017	−0.127 *	1.000			
Practiced biosecurity	0.143 *	0.046	0.141 *	0.110 *	0.050	0.084 *	−0.051	−0.180 *	0.037	−0.267 *	−0.143 *	−0.065 *	0.322 *	1.000		
Had heard of Salmonella	0.478 *	0.011	0.026	0.081	0.123 *	0.196 *	−0.198 *	−0.174 *	−0.054	−0.126 *	0.038	−0.046	−0.227 *	0.172 *	1.000	
Knowledge of Salmonella	0.343 *	−0.003	−0.066 *	−0.084 *	0.101 *	0.221 *	−0.122 *	−0.209 *	−0.057	−0.042	−0.017	−0.053	0.119 *	0.170 *	0.456 *	1.000

* Significant at *p* = 0.05. Only the ‘Heard of Salmonella’ variable was moderately correlated with ‘Experienced Salmonella’, while the ‘Knowledge of Salmonella’ was weakly predicted by the variable ‘Experienced Salmonella’. All other variables were poorly or negatively correlated with the experience of Salmonella.

**Table 4:** Univariable analysis for contamination of poultry farms with Non-Typhoidal Salmonella (NTS) in North Central Nigeria.

Variable	Category	OR (95% CI)	Chi-Square Value	*p*-Value *
Farming Experience in Years	<2 years	1.00	2.54	Ref
	2–4 years	0.87 (0.61; 1.23)		0.43
	>4–6 years	0.99 (0.69; 1.44)		0.98
	>6 years	1.15 (0.79; 1.68)		0.47
Level of education of the poultry farmer	Primary	1.00	3.90	Ref
	Secondary	0.79 (0.49; 1.26)		0.32
	Tertiary	0.91 (0.58; 1.43)		0.68
	Other forms (skill learning, etc.)	0.42 (0.15; 1.18)		0.10
Number of chickens on the farm	<200	1.00	60.09	Ref
	201–500	1.47 (1.05; 2.06)		0.03
	501–1000	2.93 (2.10; 4.11)		<0.001
	>1000	3.79 (2.45; 5.87)		<0.001
Source of feed	Multi-sourced commercial	1.00	41.28	Ref
	Bought-in concentrate and mix	1.87 (1.38; 2.54)		<0.001
	Self-compounded	0.47 (0.32; 0.70)		<0.001
Source of water	Borehole	1.00	59.83	Ref
	Pipe-borne municipal water	1.53 (1.10; 2.13)		0.01
	Dug-up well	0.42 (0.30; 0.58)		<0.001
	Stream	2.33 (1.19; 4.58)		0.01
Pen type	Standard type house (fully built)	1.00	8.81	Ref
	Dwarf block with side nets	0.90 (0.67; 1.22)		0.51
	Zinc-sided (roofing sheet) house	0.61 (0.43; 0.86)		0.005
	Other forms of buildings	0.77 (0.37; 1.61)		0.49
Management system	Deep litter	1.00	16.10	Ref
	Battery cage	1.74 (1.33; 2.28		<0.001
	Others (semi-intensive, etc.)	1.25 (0.66; 2.40)		0.49
Litter management	Good	1.00	11.13	Ref
	Poor	1.14 (0.74; 1.75)		0.59
	Fair	0.62 (0.46; 0.84)		0.002
Litter materials used	Saw dust	1.00	4.62	Ref
	Wood shavings	1.00 (0.74; 1.35)		0.99
	Sand (non-cemented floor)	0.87 (0.57; 1.33)		0.53
	Cemented floor	1.33 (0.91; 1.95)		0.14
	Other types (straw, etc.)	2.03 (0.63; 6.51)		0.23
Pen odour	Yes	1.00	0.72	Ref
	No	0.13 (0.87; 1.46)		0.36
Stocking density (chickens per square meter of available floor space)	12–14	1.00	3.59	Ref
	15–16	0.84 (0.55; 1.27)		0.40
	17–18	0.83 (0.55; 1.23)		0.35
	19–20	0.68 (0.43; 1.10)		0.12
	>20	0.64 (0.36; 1.14)		0.13
Adherence to vaccination	Yes	1.00	46.85	Ref
	No	7.43 (3.65; 15.10)		<0.001
	Partial	4.36 (2.09; 9.10)		<0.001
Implementation and adherence to biosecurity	Yes	1.00	20.84	Ref
	No	1.99 (1.30; 3.06)		0.002
	Partial	1.14 (0.72; 1.79)		0.58
Types of chickens on the poultry farm	Broiler	1.00	14.71	Ref
	Laying stock	1.87 (1.35; 2.59)		<0.001
	Other species/stock	1.07 (0.54; 2.14)		0.85
	Mixed	1.30 (0.96; 1.76)		0.09

* *p*-values were obtained through Wald test.

**Table 5:** Multivariable analysis for contamination of poultry farms with non-typhoidal Salmonella (NTS) in North Central Nigeria.

Variable	Category	Crude OR (95% CI)	Adjusted OR (95% CI)	*p*-Value *
Number of chickens on the farm	<200	1.00	1.00	Ref
	201–500	1.41 (0.95; 2.10)	1.42 (0.92; 2.20)	0.11
	501–1000	2.82 (1.92; 4.15)	2.20 (1.44; 3.37)	**<0.001**
	>1000	3.32 (2.03; 5.44)	2.17 (1.28; 3.71)	**0.004**
Source of feed	Multi-sourced commercial	1.00	1.00	Ref
	Bought concentrate and mix	1.55 (0.92; 1.92)	1.49 (0.99; 2.25)	0.07
	Self-compounded	0.54 (0.35; 0.84)	0.70 (0.42; 1.18)	0.18
Source of water	Borehole	1.00	1.00	Ref
	Pipe-borne municipal water	1.33 (0.92; 1.92)	1.49 (0.99; 2.25)	0.06
	Dug-up well	0.43 (0.29; 0.62)	0.57 (0.37; 0. 87)	**0.01**
	Stream	2.18 (1.03; 4.60)	3.31 (1.45; 7.58)	**0.005**
Litter management	Good	1.00	1.00	Ref
	Poor	1.03 (0.65; 1.64)	1.16 (0.67; 2.01)	0.59
	Fair	0.55 (0.38; 0.80)	0.67 (0.44; 1.02)	0.06
Pen odour	No	1.00	1.00	Ref
	Yes	1.26 (0.94; 1.69)	1.56 (1.12; 2.18)	**<0.01**
Adherence to vaccination (*Fowl typhoid and fowl cholera (pullorum)*)	Yes	1.00	1.00	Ref
	No	8.33 (3.49; 19.84)	5.18 (1.96; 13.66)	**<0.001**
	Partial	5.09 (2.07; 12.51)	5.10 (1.85; 14.04)	**0.002**
Implementation and adherence to biosecurity	Yes	1.00	1.00	Ref
	No	2.08 (1.26; 3.41)	1.54 (0.87; 2.72)	0.14
	Partial	1.14 (0.67; 1.94)	0.73 (0.40; 1.33)	0.31

* *p*-values were obtained through Wald test. Bold *p*-values were significant. Akaike information criterion (AIC) = 945.52; Hosmer–Lemeshow goodness of fit = Χ^2^ = 2.58; *p*-value = 0.96; area under curve (receiver operating characteristics (ROC)) = 0.72.

## Data Availability

This work is part of the PhD study of A.O.S. All data associated with this work and other components of the PhD study will be permanently archived with the Department of Veterinary Tropical Diseases, University of Pretoria, South Africa, and will be made available publicly, including the final thesis.

## Supplementary Materials

The following supporting information can be downloaded at: https://www.mdpi.com/article/10.3390/antibiotics11081121/s1. Table S1: Categorization of variables based on selected industry standards and peer-reviewed literature; File S1: Sample questionnaire for risk factor data collection in the field.
