# Supplementary material for: Risk Factors for Persistent Infection of Non-Typhoidal Salmonella in Poultry Farms, North Central Nigeria
Source: Antibiotics (Basel). 2022 Aug 18;11(8):1121. doi: 10.3390/antibiotics11081121 (PMC9405283; doi:10.3390/antibiotics11081121)
Supplement: Supplementary file 1 [file antibiotics-11-01121-s001.zip › antibiotics-1825588-supplementary.pdf]

Table S1. Categorization of Variables based on selected industry standards and peer reviewed literature.

Question	Category	Reference	Reclassification	Notes	Reference
State	State by state	No		-	-
Serial number	-	No		-	-
L .G. A	L. G. A by L. G. A	No		-	-
Gender	0 vs 1	Y	M=1<br>F=0	There 57% of women and 51% of men with a gender gap of 7.2% are involved in poultry.	The World Bank Nigeria development report (2021) (https://www.worldbank.org/en/country/nigeria/publication/nigeria-development-update-ndu).
Age of respondents	0 vs 1	Y	41 and above = 1<br>1-40 = 0	The mean age of poultry farmers is 40 years	Gender participation in commercial poultry production (http://www.lrrd.org/lrrd22/9/okoh22160)
Length in poultry farming	0 vs 1	Y	6 and above =1<br>1-5 = 0	65% of farmers have mean farming experience of 6 years	Differentials in technical efficiency among broiler farmers in Imo state Nigeria (https://www.ajol.infor/index.php/naj/article/view/196166/185183)
Education level	0 vs 1	Y	Primary &secondary =0<br>Tertiary & others higher qualifications =1	40% of poultry farm owners hold secondary school cert, 35% a university degree and 8.3% a primary school certificate.	Socio-economic factors as determinants of farm management skills (www.resarchgate.net/publication/321650666)

Farm location				Skipped, not analyzed	
Name of farm				Skipped, not analyzed	
Type of poultry	0 vs 1	Y	Broiler = 0<br>Layer & others =1		Short cycle and long cycle
Number of chickens in the farm	0 vs 1	Y	500 and above = 0<br>1-499 = 1		-
Source of feed	0 vs 1	Y	Self-compounded = 1<br>Commercial = 0	Poultry farmers prefer to use self-compounded feeds than commercial feeds	Poultry farmers preference and use of commercial and self-formulated feeds (https://www.researchgate.net/publication/223151006)
Source of water for birds	0 vs 1	Y	Borehole/tap borne = 0<br>Stream/well/ others = 1	27% of poultry farmers depend on borehole, tap water combined and 3% on depended solely well, stream or river.	quality of different water sources used in poultry and piggery farms in southeastern Nigeria (https://www.researchgate.net/publication/349180707)
Pen type	0 vs 1	Y	Standard block= 0<br>Others = 1	In commercial and semi commercial setting in developing countries, chickens are normally housed in naturally ventilated pen with additional lightning provided in form of electricity	Poultry development review (https://wwwfao.org/3/i3531e/i3531e.pdf)

System of management	0 vs 1	Y	Deep litter = 1 Battery cage = 0	There are three primary intensive control: deep litter, battery case and wire floor system.	Types of poultry management systems (https://fabioclass.com/poultry-management-systems/)
Litter material	0 vs 1	Y	Saw dust/wood shavings/sand =0 Cement floor= 1		Beddings and no beddings
Litter management	0 vs 1	Y	Good=0 Poor/fair=1	Daily grading of litter should be done.it is advisable to also use dry lime in order to keep litre dry.	Poultry litter management for better performance and production (https://www.pashudhanpraharee.com/poultry-litter-management-for-better-performance-and-production/)
Pen odour	0 vs 1	Y	Yes =1 No =0	Ammonia is the cause of pen odor and the most environmentally significant aerial pollution associated with poultry production	Poultry development review (https://wwwfao.org/3/i3531e/i3531e.pdf)
Stocking density	0 vs 1	Y	1-16 =0 17 & above = 1		Code of practice 2012, broiler production South Africa http://www.sapoultry.co.za/pdf-docs/code-practice-broilers.pdf
Adherence to Vaccination	0 vs 1	Y	Yes=1 No/partial=0	87% of poultry farmers vaccinate their chickens	An appraisal of the use of vaccination for disease prevention in poultry in Ibadan, Nigeria. (www.ajol.info/index.php/bahpa/article/view/76526)

Practice biosecurity	0 vs 1	Y	Yes = 1<br>No/partial = 0	Practice of biosecurity in the study area was high	Adoption of biosecurity for disease prevention and control by poultry farmers in Imo state, Nigeria (www.ajol.info/index.php/jafs/article/view/204206)
Ever administered fowl typhoid/ cholera vaccine	0 vs 1	Y	Yes = 1<br>No = 0	4% of farmers vaccinate chickens against fowl typhoid and fowl cholera	An appraisal of the use of vaccination for disease prevention in poultry in Ibadan, Nigeria. (www.ajol.info/index.php/bahpa/article/view/76526)
Ever heard of Salmonella infection in poultry	0 vs 1	Y	Yes = 1<br>No = 0	-	-
Ever experienced Salmonellosis	0 vs 1	Y	Yes=1<br>No=0	Large scale farms had experienced more salmonella prevalence at 33% prevalence rate	Prevalence of salmonella in chicken , farm attendants and beddings (www.researchgat.net/figure/prevalence-of-salmonella-in-chickens-farm-attendants-and-bediing -in-hawassa-and-bonga- tbi2 317032593)
If salmonella ever encountered how was it managed/controlled	0 vs 1	Y	Antibiotic/Vaccination = 1<br>Others = 0	-	Survey
Knowledge of Salmonella as a zoonotic agent	0 vs 1	Y	No knowledge = 0<br>Knowledge = 1	Majority of respondent have good knowledge about poultry diseases but not poultry zoonotic diseases	Understanding attitude, practices and knowledge of zoonotic infectious disease risks among poultry farmers in Ghana (https://onlinelibrary.wiley.com/doi/10.1002/vms3.257)

Source of knowledge of Salmonella as major zoonotic agent	0 vs 1	Y	Media = 1<br>Others = 0	Farmers with higher education level and longer experience have improved knowledge of zoonotic poultry diseases	Understanding attitude, practices and knowledge of zoonotic infectious disease risks among poultry farmers in Ghana (https://onlinelibrary.wiley.com/doi/10.1002/vms3.257)
Ever encounter mortality of chickens	0 vs 1	Y	No = 0<br>Yes = 1	A mortality rate of 1.5% or less is normal however zero mortality is the aim.	Mortality in poultry (https://agreenerworld.org/wp-content/uploads/2018/05/TAFS-8-Mortality-in-poultry-v3.pdf)
Type of mortality	0 vs 1	Y	High & moderate = 1<br>Low/normal = 0		Survey
Ever taken sample to a veterinarian/ animal health lab.	0 vs 1	Y	No = 0<br>Yes = 1	-	-
Type of Sample	-	No			Survey
What was the result?	0 vs 1	Y	Salmonella / Salmonella + others=1<br>No Salmonella = 0	-	-
What did you do after the result?	0 vs 1	Y	Sell = 1<br>Others = 0	-	Survey
What is the cost of treatment?	0 vs 1	Y	High = 1<br>Others = 1	-	Survey

Estimated cost of mortality from salmonella	0 vs 1	Y	High= 1<br>Others= 0	-	Survey
Did Salmonella affect your production?	-	No		-	Survey
Nature of effect on production	-	No		-	Survey
Profit after sales	-	No		-	Survey
Access to professional support	0 vs 1	Y	Yes= 1<br>Others= 0	Shortage of professional affects availability of support.	Challenges and prospect of poultry industry (https://www.grin.com/document/296347)

LGA = Local Government Authority; Y = Yes.

Supplementary Material File S1. Sample Questionnaire for risk factor data collection in the field

1. State -
2. Serial Number -
3. LGA -
4. Gender - A. Male, B. Female
5. Age of respondents – A. >60, B. 41-60, C. 21-40, D.<20
6. Length in Poultry Farming - <2years, B. 2-4years, C. 4-6years, D. >6years
7. Educational level – A. primary B. Secondary C. Tertiary D. others
8. Farm location -
9. Name of farm -
10. Type of poultry (commercial or backyard) – A. broilers B. Layers C. mixed D. others
11. Number of chickens in the farm – A. <200 B. 201-500 C. 501-1000 D. >1000
12. Source of feed- A. commercial feed B. concentrate mix C. self-compounded
13. Source of water for birds – A. borehole B. tap borne C. Well D. Stream E. others (describe)
14. Pen type – A. standard block B. dwarf block C. zinc type D. others
15. System of management – A. deep litter B. battery cage C. others
16. Litter material – A. Sawdust B. wood shavings C. Sand D. cement floor E. others
17. Litter management – A. Good B. Poor C. Fair
18. Pen odor – A. Yes B. No
19. Stocking density – A. 12-14/M^2 B. 14-16/M^2 C. 16-18/M^2 D. 18-20/M^2 E. >20/M^2 F. not known
20. Adherence to vaccination - A. Yes B. No C. Partial
21. Practice biosecurity - A. Yes B. No C. Partial
22. Ever administered fowl typhoid/ cholera vaccine - A. Yes B. No
23. Ever heard of salmonella infections in poultry - A. Yes B. No
24. Ever experienced salmonella infection on farm - A. Yes B. No C. Don’t Know

25. If salmonella ever encountered how was it managed/controlled – A. antibiotics B. Vaccination C. antibiotics and vaccination D. culling and sale E. others
26. Knowledge of salmonella as a zoonotic agent - A. Yes B. No
27. Source of knowledge of Salmonella as a zoonotic agent A. electronic media B. print media C. extension agent D. vet/animal health officer E. other farmers F. hospital
28. Ever encountered mortality of chickens - A. Yes B. No
29. Type of mortality A. high B. Moderate C. Low D. normal occurrence
30. Ever taken samples to a veterinarian/animal health lab - A. Yes B. No
31. What type of samples – A. feces B. Egg C. whole bird D. all E. others
32. What was the result – A. salmonella B. salmonella and another infection C. other
33. What did you do after the result? A. Treat B. Sell C. others
34. What is the cost of treatment – A. high B. Moderate C. Low D. others
35. Estimated cost of mortality from salmonella – A. high B. Moderate C. Low D. others
36. Did salmonella affect your production - A. Yes B. No
37. Nature of effect on production: A. high B. Moderate C. Low D. others
38. Profit after sales - A. Yes B. No
39. Access to professional support - A. Yes B. No C. Not always D. others
